# KLK8 promotes the proliferation and metastasis of colorectal cancer via the activation of EMT associated with PAR1

**DOI:** 10.1038/s41419-021-04149-x

**Published:** 2021-09-22

**Authors:** Qing Hua, Zhirong Sun, Yi Liu, Xuefang Shen, Weiwei Zhao, Xiaoyan Zhu, Pingbo Xu

**Affiliations:** 1 Department of Anesthesiology, Shanghai Cancer Center, Fudan University, Shanghai, China; 2 Department of Oncology, Shanghai Medical College, Fudan University, No. 270 Dong an Road, 200032 Shanghai, China; 3 Department of Anesthesiology, Zhongshan Hospital, Fudan University, 200032 Shanghai, China; 4 Department of Integrated Therapy, Fudan University Shanghai Cancer Centre, Shanghai, China; 5 Department of Physiology, Navy Medical University, 800 Xiangyin Road, 200433 Shanghai, China

**Keywords:** Colorectal cancer, Oncogenes, Tumour biomarkers

## Abstract

Kallikrein-related peptidase 8 (KLK8) acts as an oncogene or anti-oncogene in various tumours, and the abnormal expression of KLK8 is involved in the carcinogenesis of several tumours. However, the role of KLK8 in colorectal cancer (CRC) and the underlying mechanism remain largely unclear. In this study, the carcinogenic effect of KLK8 was determined via CCK-8 and colony formation assays in vitro and a xenograft model in nude mice in vivo. The metastasis-promoting effect of KLK8 was investigated with transwell migration and invasion assays and wound-healing assay in vitro and a metastasis model in nude mice in vivo. Bioinformatics analyses and mechanistic experiments were conducted to elucidate the molecular mechanism. Herein, we reported that KLK8 had a promotive effect on the proliferation, migration and invasion of RKO and SW480 cells. Epithelial−mesenchymal transition (EMT) played an important role in the promotive effects of KLK8 on CRC. In addition, protease-activated receptor-1 (PAR-1) antagonist SCH79797 but not protease-activated receptor-2 (PAR-2) antagonist FSLLRY-NH2 attenuated the proliferation, migration and invasion of KLK8-upregulated RKO and SW480 cells. PAR-1 antagonist SCH79797 reduced the tumour volume of xenograft model and decreased the metastatic nodules in the livers of metastasis model. Furthermore, SCH79797 could reverse the positive impact of KLK8 on the EMT process in CRC both in vitro and in vivo. Taken together, these findings demonstrated for the first time that KLK8 promoted EMT and CRC progression, and this effect might be, at least partly mediated by PAR1-dependent pathway.

## Introduction

Colorectal cancer (CRC) is one of the most common cancers in both sexes [1]. According to 2020 cancer statistics, approximately 147,950 cases of CRC will be newly diagnosed and 53,200 CRC-related deaths will occur in the United States in 2020 [2]. Although the overall survival of CRC patients has increased during the past two decades due to chemotherapy, immunotherapy and targeted therapy, metastasis remains the major obstacle in the treatment and prognosis of CRC because of its local invasion and distant metastasis [3, 4].

The human kallikrein-related peptidase family (KLKs), which is composed of 15 serine proteases (KLK1−KLK15), is a group of trypsin- and chymotrypsin-like serine proteases that share a similar homology to the parent human tissue kallikrein (KLK1) [5, 6]. Abnormal expression of KLKs is relevant to cancer cell proliferation, invasion and metastasis [6–9] in ovarian cancer, gastric cancer and non-small cell lung cancer. To gain insight into the effects of KLKs on the progression and prognosis of CRC, the present study investigated the expression profiles of KLKs in CRC using TCGA and GTEx databases and found that KLK1, KLK6, KLK8, KLK10, KLK11 and KLK12 were highly expressed in tumour tissues compared with normal tissues. Then, Kaplan–Meier survival analysis and log-rank tests were used to further investigate the relationships between these highly expressed KLKs and the prognosis of CRC and found that only KLK6, KLK8 and KLK10 predicted poor prognosis in CRC. Previous studies have reported the clinical significance and underlying mechanisms of KLK6 [10–12] and KLK10 [13–15] in CRC [16–18]. However, the role of KLK8 in CRC remains largely unknown.

To explore the mechanisms by which KLK8 promotes CRC progression, the gene expression in CRC tissues with high KLK8 expression and those with low KLK8 expression was analysed by gene set enrichment analysis (GSEA) based on the TCGA database. Among all the enriched pathways that were differentially expressed according to the diverse KLK8 expression levels in GSEA, epithelial−mesenchymal transition (EMT), a key step in enhancing cancer cell invasion and metastasis [19–22], attracted our attention. EMT refers to the reprogramming of epithelial cells to acquire a mesenchymal-like phenotype that can limit total surgical resection and induce therapeutic resistance, finally leading to tumour recurrence [23].

As a secretory serine protease, KLK8 mainly hydrolyses substrate proteins, such as extracellular matrix or cell membrane surface proteins, and plays an important role in tissue reconstruction and cell function regulation [24, 25]. KLKs were recently shown to regulate cell signalling by cleaving and activating protease-activated receptors (PARs), which have been implicated in many cellular effects, including hypoxia/reoxygenation (H/R) injury and hypertrophy development [5, 24, 26, 27]. Whether KLK8 activates PAR1/2 signalling in CRC has not been reported. Previous studies have shown that the activation of PAR1/2 could induce EMT in lung cancer cells, alveolar epithelial cells and mammary epithelial cells [28–30]. Therefore, we hypothesized that KLK8 might participate in the EMT of CRC to promote metastasis by activating PARs.

In this study, we first identified the correlation between the expression of KLK8 and the clinicopathologic parameters of CRC patients and then investigated the effects of KLK8 on the proliferation, migration and invasion of CRC cells. Finally, we explored the effects of KLK8 on EMT and the underlying mechanisms both in vitro and in vivo.

## Materials and methods

### Patients and specimens

A total of 350 CRC tissue samples and their matched adjacent noncancerous mucosal samples were obtained from CRC patients who underwent surgery at Fudan University Shanghai Cancer Center (Fudan Center) between June 2012 and December 2013. Ten adenoma samples, 16 metastatic adenocarcinoma samples and ten normal colon tissue samples were collected. All the diagnoses were confirmed by two pathologists. Patients with at least a 5-year follow-up were included in this study. All the specimens were acquired after written informed consent was provided following procedures approved by the Ethics Committee of Fudan University Shanghai Cancer Center. Tumour staging was determined according to the AJCC Cancer Staging Manual. Nine tissue microarrays were constructed.

### The Cancer Genome Atlas Analysis and GSEA

TCGA colon cancer (COAD) consisted of 275 CRC samples and 41 normal samples. The 308 normal samples in GTEx database were used as complement. GEPIA (http://gepia.cancerpku.cn/) and USUC Xena Cancer Genomics Browser (https://xenabrowser.net/datapages/?dataset=TCGA) were used to determine the differentially expressed genes between the normal colorectal samples and CRC samples. The correlation among the KLK expression level, the overall survival (OS) time and the disease-free survival (DFS) time were calculated using microarray data from a COAD cohort (GSE39582 database, *n* = 557) by setting the online Kaplan–Meier plotter tool for a 50% cut-off to separate patients into high and low gene expression groups. To elucidate the mechanisms underlying the role of KLK8 in CRC, gene set enrichment analysis (GSEA) was performed on the Broad Institute Platform. Hallmark gene set collection was used to identify relative signalling pathways of KLK8 from the control and KLK8 overexpression groups according to the genes presenting the strongest enrichment scores. The enrichment score (ES) was calculated to reflect the degree to which KLK8 is overrepresented at the extremes (top or bottom) of the entire ranked list. The statistical significance (nominal *P* value) of the ES was estimated by using an empirical phenotype-based permutation test procedure that preserves the complex correlation structure of the gene expression data. The ES for each gene was normalized to account for the size of the set, yielding a normalized enrichment score (NES) [31, 32].

### Animal studies

Male 4- to 6-week-old BALB/c nude mice were used to conduct all the in vivo studies. The mice were obtained from Shanghai SLAC Laboratory Animal Co. (Shanghai, China) and housed in a room with a controlled temperature with free access to food and water under a natural day/night cycle. All the animal protocols were approved by the Ethical Committee of Fudan University Shanghai Cancer Center and conformed to the principal guidelines of the Guide for the Care and Use of Laboratory Animals (8th edition, National Academies Press).

For the xenograft model, the mice were subcutaneously inoculated with 200 µL RKO-Lv-Control or RKO-Lv-KLK8 cell suspension (1 × 10^7^ cells/mL) in the right flanks. The tumour volume was measured once every 2 days and calculated as volume = (length × width^2^)/2. When the tumour reached 50 mm^3^, the mice were divided into three groups (Lv-Control group, Lv-KLK8 group and Lv-KLK8 + PAR1 inhibitor group). The mice in the Lv-KLK8 + SCH79797 group were administered 25 µg/mg/i.p. SCH79797. Twenty-five days after tumour cell inoculation, the mice were euthanized. The tumours were collected and weighed, and the tumours were collected for further analysis. The mice were humanely sacrificed by CO_2_ inhalation at 30% vol/min when they met the following humane-endpoint criteria: prostration, significant body weight loss, difficulty breathing, rotational motion and body temperature drop.

For the metastasis model, the nude mice were randomly allocated into three groups (*n* = 6) to study liver metastasis. The mice were anaesthetized by intraperitoneal administration of mixed ketamine (70 mg/kg) and xylazine (10 mg/kg). A 1-cm incision was made on the upper left lateral abdomen, and then, the spleen was pulled out of the abdominal cavity. A total of 200 µL RKO-Lv-Control or RKO-Lv-KLK8 cells (2 × 10^6^) were slowly injected under the capsule of the spleen using an insulin syringe. The injection time was approximately 3 min, and the spleen was swollen and whitened. After the injection, a 75% alcohol cotton ball was used to compress the injection site for 15 min. The spleen was then removed, and the abdominal wall was sutured. After 7 days, the mice injected with RKO-Lv-KLK8 cells were divided into two groups (Lv-KLK8 group and Lv-KLK8 + PAR1 inhibitor group, *n* = 6), and the mice in the Lv-KLK8 + PAR1 inhibitor group were administered 25 µg/mg/i.p. SCH79797. The mice in the liver metastasis model were killed 28 days after the operation. The liver was collected, fixed in 10% formalin and weighed. Metastatic nodules were identified by colour and appearance and counted under a dissecting microscope. Metastatic liver nodules were further confirmed via haematoxylin and eosin (H&E) staining.

### Cell culture and stable transfection

The human CRC cell lines RKO and SW480 were obtained from Type Culture Collection of the Chinese Academy of Sciences (Shanghai, China). The RKO and SW480 cells were cultured in Dulbecco’s modified eagle medium (DMEM) (Invitrogen). All the media were supplemented with 10% foetal bovine serum (Gibco, USA) and 1% penicillin/streptomycin (Invitrogen). To establish cell lines stably overexpressing KLK8, a pcDNA3.1-KLK8 plasmid for KLK8 gene overexpression and its negative control were designed and synthesized by Shanghai Genechem Co. (Shanghai, China). RKO and SW480 cells were stably transfected using Lipofectamine 3000 (Invitrogen, Carlsbad, CA) according to the manufacturer’s instructions, and the transfection efficiency was assessed using western blot assays.

### Transient transfection

The siRNAs for KLK8 were designed and synthesized by Genechem Co. (Shanghai, China). The target sequences for human KLK8 siRNA and plakoglobin siRNA are as follows: 5ʹ-TGGAGGACCACAACCATGATCTGAT-3ʹ and 5ʹ-CCCTCGTGCAGATCATGCGTAACTA-3ʹ, respectively. Negative control siRNA was scrambled sequence without any specific target: 5ʹ-TTCTCCGAACGTGTCACGT-3ʹ. Transfection of siRNA in RKO and SW480 cells was performed by the XfectTM RNA transfection reagent (Takara) according to the manufacturer’s instructions, and the transfection efficiency was assessed using western blot assays.

### Western blot analysis

RKO and SW480 cells were lysed, and the proteins were extracted following standard protocols. The proteins were separated by SDS-polyacrylamide gel electrophoresis and subjected to western blot analyses. The protein bands were detected by the chemiluminescence method. Specific primary antibodies against KLK8 (Abcam, ab150395, 1/1000), E-cadherin (ProteinTech, 20874-10-AP, 1/1500), N-cadherin (ProteinTech, 22018-1-AP, 1/1000), vimentin (ProteinTech, 10366-1-AP, 1/1000) and occludin (ProteinTech, 27260-1-AP, 1/2000) were used. β-Actin (Santa Cruz, sc-47778, 1/5000) was used as a loading control. The chemiluminescent signals were detected with a chemiluminescence imaging system and quantified by ImageJ software (v1.37).

### Immunohistochemical (IHC) staining

Immunohistochemical (IHC) staining of KLK8 was performed using a primary antibody against KLK8 (Abcam, ab150395, 1/150) according to the manufacturer’s instructions. Immunohistochemistry was performed on tissue microarray slides to study protein expression in clinical specimens. Staining was independently examined by two experienced investigators blinded to the clinical characteristics of the patients. The score for KLK8 staining was based on the integrated staining intensity and the percentage of positive cells. Staining intensity was scored as follows: 0 = no colour; 1 = light yellow; 2 = light brown; and 3 = dark brown. The proportion of immune-positive tumour cells (number of positively labelled tumour cells / number of total tumour cells) was scored as follows: 0, <5% positive cells; 1, 6−25% positive cells; 2, 26−50% positive cells; 3, 51−75% positive cells; and 4, >75% positive cells. The comprehensive score was the product of staining intensity and average proportion of positive cells and expressed as follows: 0, negative staining, marked −; 1–4, weak expression, marked +; 5–8, moderate expression, marked ++ and 9–12, strong expression, marked +++. All the percentages/numbers of positive cells were expressed as the average of six randomly selected microscopic fields.

### Immunofluorescence staining

RKO and SW480 cells treated with the PAR1 antagonist SCH79797 (100 µM) or the PAR2 antagonist FSLLRY-NH2 (150 nM) were incubated in 4% paraformaldehyde solution. After washing in PBS three times, the cells were blocked with 10% BSA solution at room temperature. Then, E-catenin (ProteinTech, 20874-10-AP, 1/200) and vimentin (ProteinTech, 10366-1-AP, 1/200) primary antibodies were added at the same time and incubated for 24 h at 4 °C. Following incubation with 488-conjugated secondary anti-mouse and 594-conjugated anti-rabbit IgG antibodies, the cells were labelled with 4',6-diamidino-2-phenylindole (DAPI), and images were captured.

### Cell counting kit-8 (CCK-8) assay

The pretreated cells were seeded into a 96-well plate. RKO and SW480 cells were incubated with CCK-8 reagent (Dojindo, Japan) at 37 °C for 1 h, and the absorbance was measured at 450 nm using a microplate reader (BioTek, Vermont, USA) for the appropriate time (1, 2, 3, 4 or 5 days).

### Colony formation

Log phase RKO and SW480 cells were collected, the cell suspension density was adjusted to 500 cells/well, and the cells were incubated at 37 °C and exposed to 5% CO_2_. After 24 h, the PAR1 antagonist SCH79797 (100 µM) or the PAR2 antagonist FSLLRY-NH2 (150 nM) was added and incubated for 48 h. Next, the medium containing SCH79797 or FSLLRY-NH2 was replaced with fresh complete DMEM. After 14 days, the colonies were counted visually, with >50 cells/colony considered a clone. The colonies were washed twice with PBS and then fixed with 70% methanol. After 20 min of incubation, the colonies were rinsed twice with PBS buffer and stained with crystal violet (0.2%) for 30 min. Images of each well were taken by an inverted fluorescence microscope.

### H&E staining

The liver tissues embedded in paraffin were deparaffinized and stained with haematoxylin for 10 min. After treatment with hydrochloric acid alcohol solution and ammonium hydroxide for 30 s, the samples were stained with eosin for 3 min. Increased concentrations of alcohol were used to dehydrate the sections. Next, the sections were treated with xylene three times for 3 min each. Finally, neutral balsam was used for section mounting.

### Wound-healing assay

Cells were cultured in six-well plates overnight. A sterile P200 pipet tip was used to create a straight scratch in the cell monolayer. After washing twice with PBS buffer, the plate was incubated with fresh complete DMEM containing the PAR1 antagonist SCH79797 (100 µM) or the PAR2 antagonist FSLLRY-NH2 (150 nM) for 48 h. The plate was imaged at 0 and 48 h by an inverted fluorescence microscope. All of the experiments were repeated three times.

### Transwell migration and invasion assay

Cells suspended in complete medium were cultured in the upper chamber of 8.0-μm pore Transwells (Corning-Costar, Cambridge, MA, USA) precoated without (for migration) or with (for invasion) Matrigel (BD Biosciences, San Jose, CA, USA), and the lower chamber was filled with 800 µL complete medium. After 24 h of incubation, the PAR1 antagonist SCH79797 (100 µM) or the PAR2 antagonist FSLLRY-NH2 (150 nM) was added to the upper chamber and incubated for 48 h. Then, the medium with drugs in the upper chamber was replaced with serum-free medium, and the medium in the lower chamber was replaced with 20% FBS-complete medium. After incubation for the indicated times, noninvading cells on the upper membranes were carefully cleared. The migrated or invaded cells on the lower membranes were fixed with 95% ethanol for 20 min and stained with 0.1% crystal violet. Then, the cells attached to the lower surface of the chamber in five random fields were counted under a microscope at ×200 magnification.

### Statistical analysis

The data are expressed as the means ± SEM from at least three experiments. All the statistical analyses were performed using SPSS 13.0 (SPSS Inc.). Independent samples *t* tests were used to compare individual data with the control values, and one-way ANOVA was performed to compare the data of multiple groups. The Kaplan–Meier estimation method was used for overall survival analysis, and a log-rank test was used to compare differences. Analysis of variance was utilized to analyse significant differences between groups under different conditions. *P* < 0.05 was considered to be statistically significant. (*P < 0.05, **P < 0.01, ***P < 0.001, ^#^*P* < 0 .05, ^##^*P* < 0.01).

## Results

### KLK8 is upregulated in colon cancer and correlates with poor prognosis

**Fig. 1:**
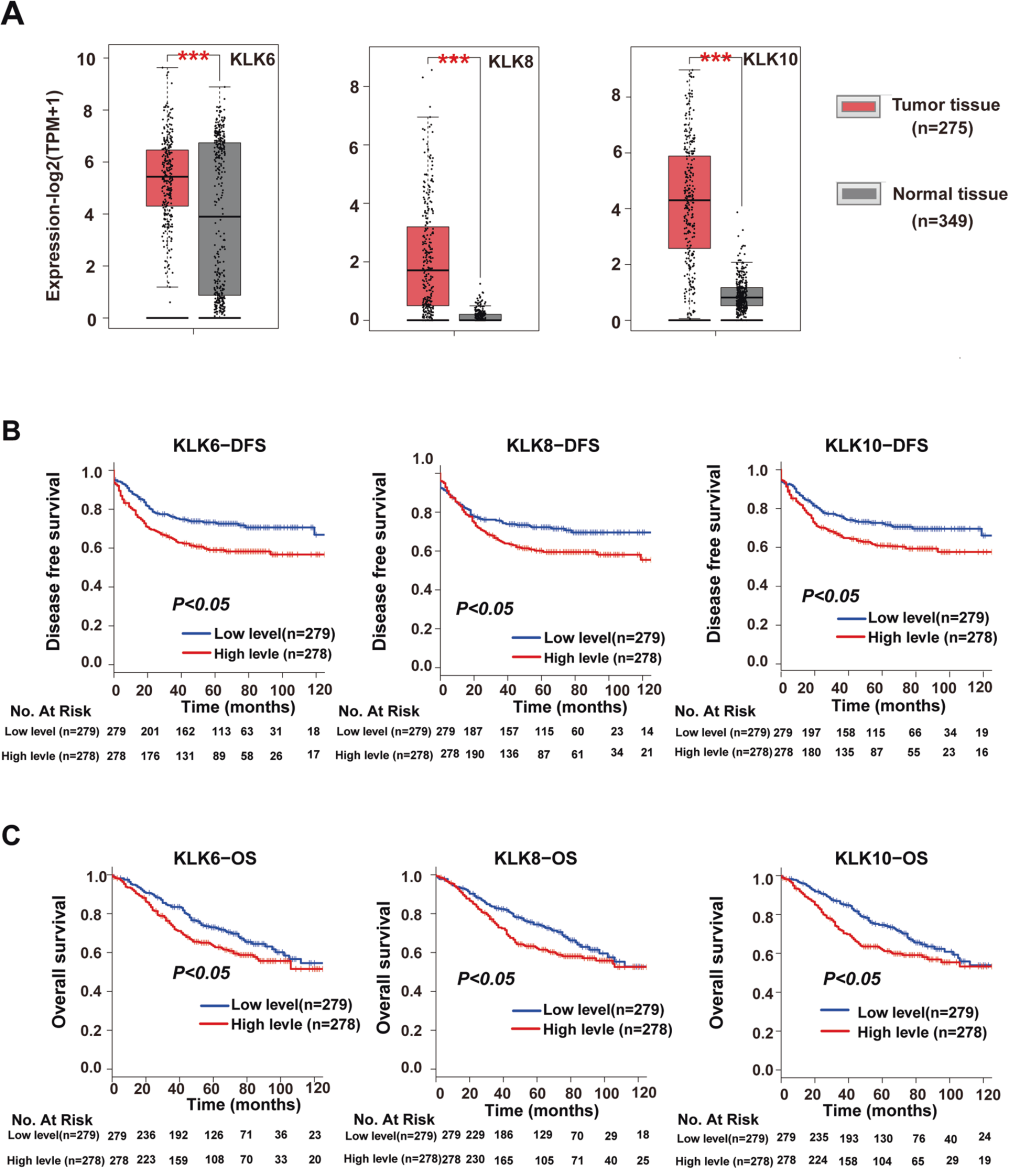
The role of KLKs in the CRC. **A** The expression of KLK6, KLK8 and KLK10 in CRC tissues (*n* = 275) and normal tissues (*n* = 349) analysed in the TCGA and GTEx databases. **B**, **C** Disease-free survival (DFS) (**B**) and overall survival (OS) (**C**) were compared between patients with low and high KLK6, KLK8 and KLK10 expression in the GSE39582 database. ***P < 0.001.

To explore the roles of KLKs in the progression and prognosis of CRC, we first analysed the expression profiles of KLKs in CRC using the TCGA-COAD cohort and GTEx databases in GEPIA (http://gepia2.cancer-pku.cn/#analysis). We found that the expression levels of KLK1, KLK6, KLK8, KLK10, KLK11 and KLK12 were elevated in CRC tissue samples compared to normal tissue samples (Fig. 1A and Supplementary Fig. S1A, *P* < 0.0001). Then, we evaluated the relationship among these highly expressed KLKs and patient outcomes. Kaplan–Meier curve analysis of the GSE39582 database indicated that higher expression of KLK6, KLK8 and KLK10 in CRC was correlated with shorter OS and DFS rates (Fig. 1B, C and Supplementary Fig. S1A, C, *P* < 0.05). Median OS for KLK6 high-expression patients was 47 months in contrast to 53 months in the KLK6 low-expression group; median DFS for KLK6 high-expression patients was 34 months in contrast to 48 months in the KLK6 low-expression group; median OS for KLK8 high-expression patients was 47 months in contrast to 54 months in the KLK8 low-expression group; median DFS for KLK8 high-expression patients was 36 months in contrast to 48 months in the KLK8 low-expression group; median OS for KLK10 high-expression patients was 46 months in contrast to 54 months in the KLK10 low-expression group; median DFS for KLK10 high-expression patients was 36 months in contrast to 48 months in the KLK10 low-expression group. As previous studies have reported the clinical significance and underlying mechanisms of KLK6 [10–12] and KLK10 [13–15] in CRC [16–18], we focused on the effects of KLK8 on the development of CRC.

**Fig. 2:**
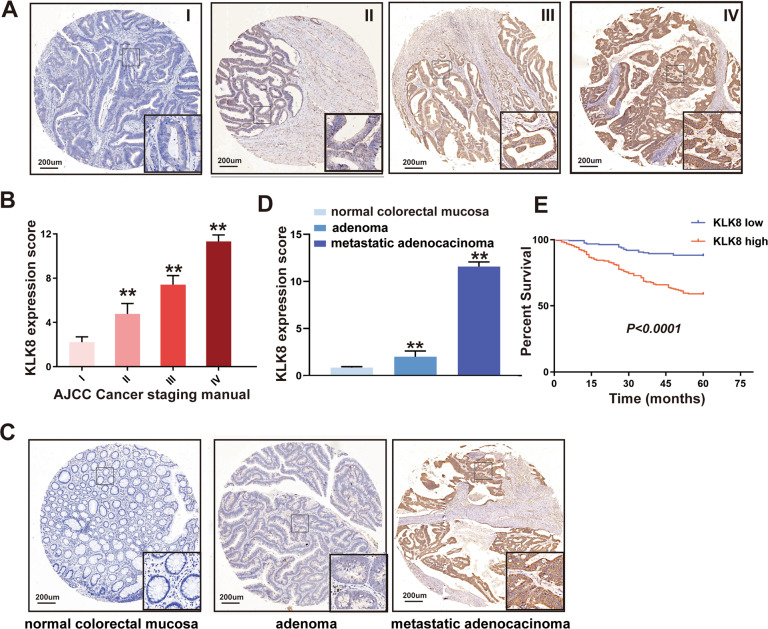
Kallikrein-related peptidase 8 (KLK8) gene is highly expressed in colorectal cancer and correlates with poor prognosis. **A** KLK8 expression in colorectal cancer tissues at different stages, from I to IV based on AJCC Cancer Staging Manual, were measured using IHC. KLK8 expression scores were shown in (**B**). **C** KLK8 expression in normal colon tissue, adenoma and metastatic adenocarcinoma was measured using IHC. KLK8 expression scores were shown in (**D**). **E** Comparison of the percent of survival of patients with high-level KLK8 (KLK8-High) and low level KLK8 (KLK8-Low) expression using the Kaplan−Meier method (*n* = 350). *P* < 0.0001. **P < 0.01 versus normal colon.

First, we analysed the expression of KLK8 in CRC patients using four independent public datasets from Oncomine (https://www.oncomine.org/resource/main.html) and found that KLK8 expression was elevated in CRC tissue samples compared to normal tissue samples (Supplementary Fig. S2, *P* < 0.0001). Then, we performed IHC staining on 350 CRC patient samples, which confirmed that KLK8 expression was higher in the late stage of colon cancer (AJCC Cancer Staging Manual, 7th edition) (Fig. 2A, B, *P* < 0.001). Moreover, the expression of KLK8 increased gradually in normal colon tissue, adenoma and metastatic adenocarcinoma (Fig. 2C, D). Then, we evaluated the relationship between KLK8 expression and patient outcomes in our samples and found that higher KLK8 expression in the tumour tended to confer a significantly poorer prognosis in 350 CRC patients who underwent radical colectomy at Fudan University Shanghai Cancer Center (Fudan Center) (Fig. 2E). The 5-year survival rate of CRC patients in the KLK8-high group was 83.654% and 60.511% in the KLK8-low group. These results indicated that KLK8 may act as a tumour promoter in colon cancer and predict an adverse prognosis.

### KLK8 overexpression enhanced proliferation, migration and invasion of colorectal cancer whereas KLK8 knockdown showed the attenuated effect in vitro

**Fig. 3:**
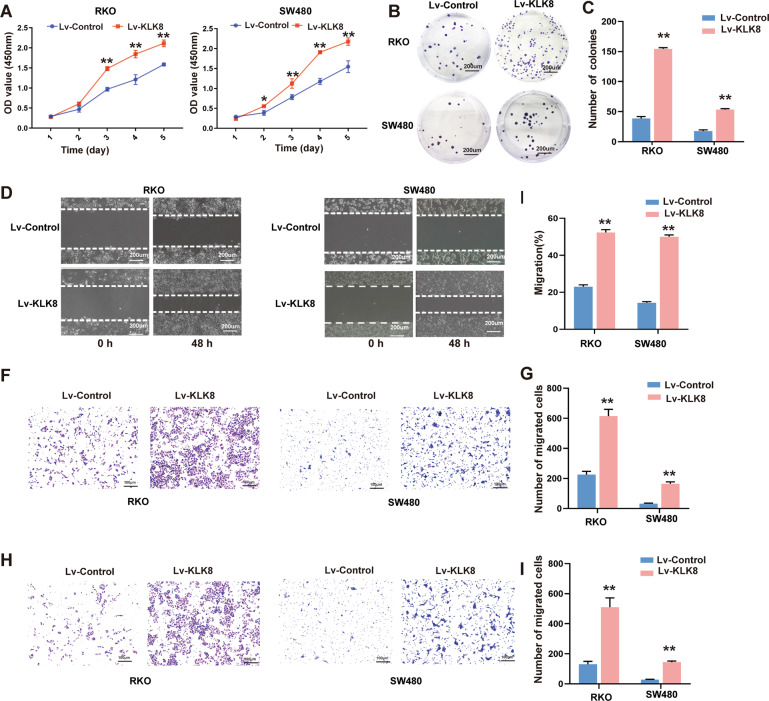
KLK8 overexpression promotes the proliferation, migration and invasion of CRC cells in vitro. KLK8 overexpression was induced with recombinant Lentivirus infection (Lv-KLK8) in human colorectal cancer cell lines RKO and SW480, and an empty adenovirus served as control (Lv-Control). **A** CCK8 assay was used to assess cell proliferation. **B** Colony formation assays for the proliferative ability of Lv-control or Lv-KLK8-transfected CRC cells. The number of colonies was shown in (**C**). **D**, **E** Wound-healing assay was conducted to assay the migration of CRC cells. The wound space was photographed at 0 and 48 h. The cell migration ability was evaluated by measuring the distance between the advancing margins of cells. **F**−**I** Cell migration and invasion were determined by using transwell migration and invasion assays. All of the data are presented as the mean ± SEM from three independent experiments. *P < 0.05; **P < 0.01.

Abnormal cell proliferation, migration and invasion are characteristics of human malignant tumours [17, 33]. We first established stable KLK8 overexpression in RKO and SW480 cells to ascertain the role of KLK8 in controlling the proliferation and metastatic potential of CRC. The proliferative activity was assessed by CCK-8 and colony formation assays, and the migratory and invasive activities were assessed by wound-healing assays and transwell migration and invasion assays, respectively. The efficacy of KLK8 overexpression in the two cell lines was confirmed by western blot analysis (Supplementary Fig. S3A). As shown in Fig. 3A, a significant promotion of cell proliferation was observed in the KLK8-overexpression group compared to the control group. In addition, the number of cell colonies was significantly increased in both RKO and SW480 CRC cells overexpressing KLK8 (Fig. 3B, C). Moreover, we elucidated the effect of KLK8 overexpression on the metastasis of CRC cells. The wound-healing assay indicated that cells with higher KLK8 expression showed significantly more rapid wound closure than their respective controls (Fig. 3D, E). Furthermore, transwell assays showed that the overexpression of KLK8 markedly enhanced the migration (Fig. 3F, G) and invasion (Fig. 3H, I) abilities of RKO and SW480 cells.

**Fig. 4:**
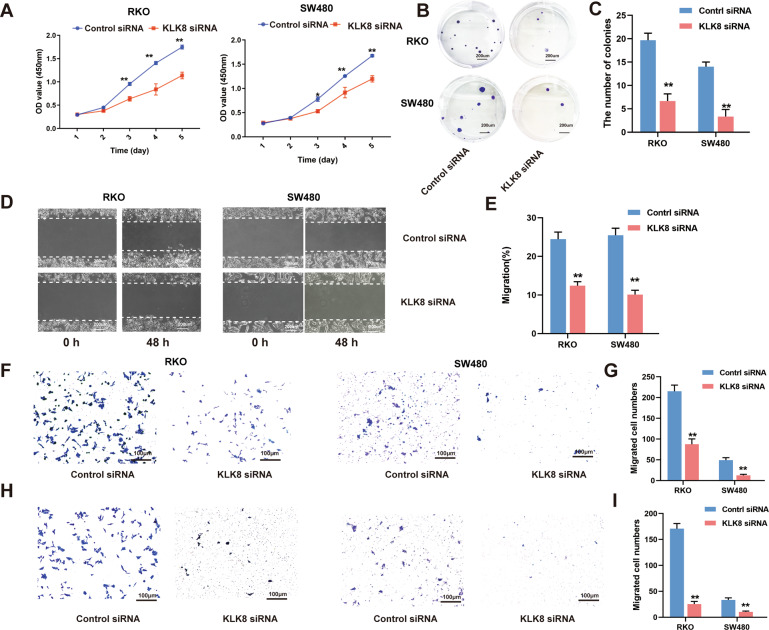
KLK8 knockdown inhibited the proliferation, migration and invasion of CRC cells in vitro. RKO and SW480 cells were transfected with control siRNA or KLK8 siRNAs. **A** CCK8 assay was used to assess cell proliferation. **B** Colony formation assays for the proliferative ability of Control siRNA or KLK8 siRNA-transfected CRC cells. The number of colonies was shown in (**C**). **D**, **E** Wound-healing assay was conducted to assay the migration of CRC cells. The wound space was photographed at 0 and 48 h. The cell migration ability was evaluated by measuring the distance between the advancing margins of cells. **F**−**I** Cell migration and invasion were determined by using transwell migration and invasion assays. All of the data are presented as the mean ± SEM from three independent experiments. *P < 0.05; **P < 0.01.

Then, we further observed the effect of siRNA-mediated KLK8 knockdown on colon cancer cell proliferation, migration and invasion. The efficacy of KLK8 knockdown in the two cell lines was confirmed by western blot analysis (Supplementary Fig. S3B). It was found that KLK8 knockdown led to a significant inhibition of cell proliferation in both RKO and SW480 cells (Fig. 4A). The number of cell colonies was also significantly decreased in KLK8 siRNA-treated RKO and SW480 cells compared with control siRNA-treated CRC cells (Fig. 4B, C). In the migration and invasion experiments, the wound-healing assay indicated that KLK8 siRNA-treated RKO and SW480 cells showed significantly slower wound closure than control siRNA-treated (Fig. 4D, E). Furthermore, transwell assays showed that the low expression of KLK8 markedly inhibited the migration (Fig. 4F, G) and invasion (Fig. 4H, I) abilities of RKO and SW480 cells.

Taken together, these findings suggest that KLK8 might be a favourable factor for the proliferation, migration and invasion of CRC cells.

### KLK8 facilitated EMT in colon cancer cells in vitro

**Fig. 5:**
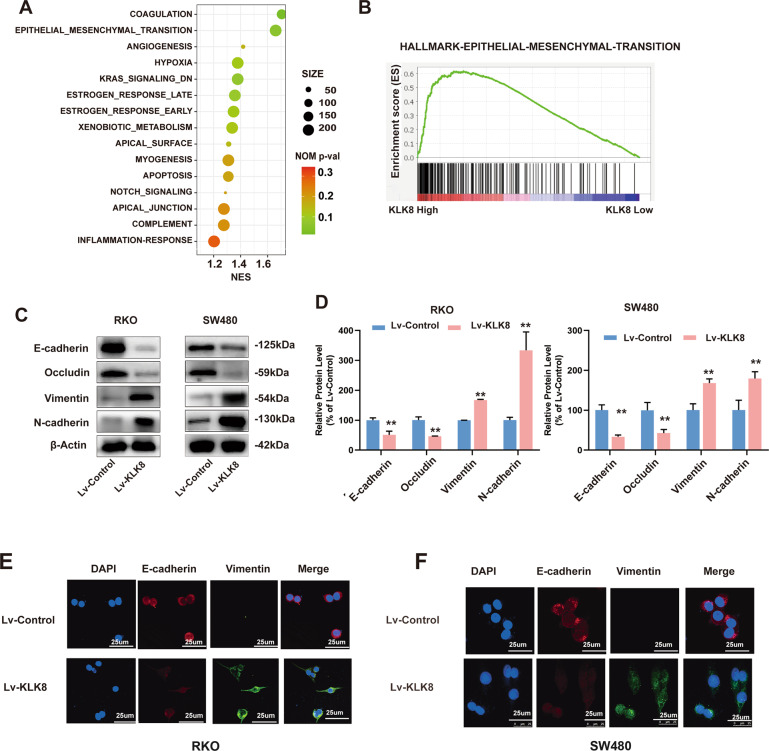
KLK8 facilitated EMT in colon cancer cells in vitro. **A** Gene sets enriched in the transcriptional profiles of tumours belonging to the top KLK8 high-expression group, compared with the bottom-expression group in the TCGA dataset. Shown are the NES (normalized enrichment score) values for each pathway using the Hallmark gene sets. The functional annotations of KLK8-positive and -negative expression in CRC was predicted. **B** Positive correlation between colorectal cancer EMT and KLK8 expression was shown using the TCGA database (*n* = 545). **C** Western blotting analysis showed that KLK8 decreased the expression levels of the epithelial cell markers E-cadherin and occludin and increased the mesenchymal markers N-cadherin and vimentin in KLK8-overexpression RKO and SW480 cells. **D** Quantification of the protein expression of E-cadherin, N-cadherin, vimentin and occludin represented in (**C**). **E**, **F** Immunofluorescence of E-cadherin and vimentin in RKO and SW480 cells overexpressing KLK8 or not. All of the data are presented as the mean ± SEM from three independent experiments. **P < 0.01.

EMT is a central driver of epithelium-derived tumour malignancies, which triggers the dissociation of carcinoma cells from primary carcinomas that subsequently migrate and disseminate to distant sites [34]. Gene set enrichment analysis (GSEA) using TCGA datasets showed that KLK8 expression is associated with the regulation of EMT in CRC tissues (Fig. 5A, B). To further assess the relationship between KLK8 and EMT in CRC, we investigated the expression of the EMT-associated markers E-cadherin, N-cadherin, occludin and vimentin. E-cadherin and occludin, hallmarks of epithelial cells, were significantly decreased in KLK8-overexpressing RKO and SW480 cells (Fig. 5C, D). Concomitantly, N-cadherin and vimentin, which are mesenchymal cytoskeletal proteins, were markedly increased in KLK8-overexpressing RKO and SW480 cells (Fig. 5C, D). In addition, we performed immunofluorescence to analyse the protein expression of E-cadherin and vimentin in CRC cell lines (Fig. 5E, F). We found that both RKO and SW480 cells overexpressing KLK8 had decreased expression of E-cadherin and increased expression of vimentin compared with control cells. Collectively, these results indicate that KLK8 promotes CRC cell metastasis by inducing EMT.

### PAR1 inhibition suppresses the proliferation, migration and invasion of colorectal cancer cells induced by KLK8 in vitro

**Fig. 6:**
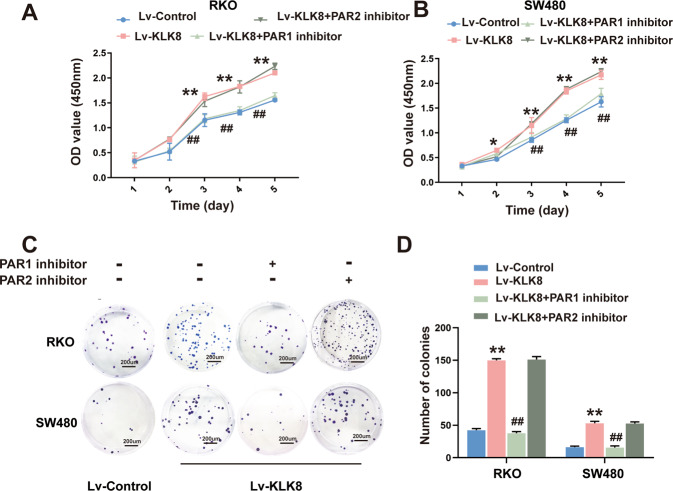
PAR1 inhibition suppresses the proliferation in colorectal cancer cells induced by KLK8 in vitro. KLK8-overexpressed RKO and SW480 cells were treated with PAR1 antagonist SCH79797(100 μM) or PAR2 antagonist FSLLRY-NH2 (150 nM). **A**, **B** CCK8 showed the proliferation rate of CRC cells. **C** Colony formation assay for the proliferative ability of Lv-control or Lv-KLK8-transfected CRC cells treatment with PAR1 antagonist SCH79797 or PAR2 antagonist FSLLRY-NH2. **D** Histogram represents the statistical analysis of (**C**). All of the data are presented as the mean ± SEM from three independent experiments. *P < 0.05, **P < 0.01 vs. Lv-control; 0.05, ^##^*P* < 0.01 vs. Lv-KLK8.

KLKs were recently shown to regulate cell function by cleaving and activating members of the PAR family [26, 27]. Our previous study found that both PAR1 and PAR2 inhibitors and knockdown could significantly reverse KLK8-induced hypertrophy in cardiomyocytes [24]. We investigated whether PAR1 and PAR2 contributed to the proliferation and metastasis of CRC induced by KLK8. As shown in Fig. 6A, B, the CCK-8 assay showed that the proliferative activity of RKO and SW480 cells overexpressing KLK8 was reversed by the PAR1 antagonist SCH79797 but not the PAR2 antagonist FSLLRY-NH2. Moreover, the colonies of the KLK8-overexpression groups were decreased when using the PAR1 antagonist SCH79797 (Fig. 6C, D).

**Fig. 7:**
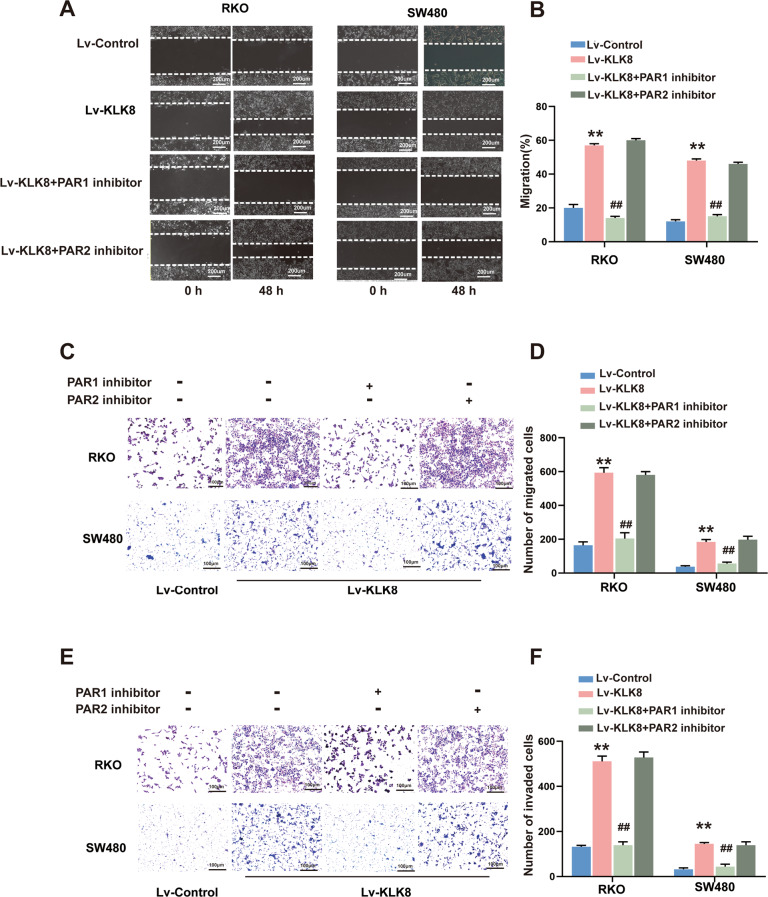
PAR1 inhibition suppresses the migration and invasion of colorectal cancer cells induced by KLK8 in vitro. **A**, **B** Wound-healing assay was conducted to assay the migration of CRC cells. **C**−**F** Transwell assays showing migration/invasion of cells. Cells invading through uncoated inserts and Matrigel-coated inserts were stained, ×400. All of the data are presented as the mean ± SEM from three independent experiments. **P < 0.01 vs. Lv-control; ^##^*P* < 0.01 vs. Lv-KLK8.

We then elucidated the effect of PAR1 and PAR2 on the metastasis of CRC cells overexpressing KLK8. As shown in Fig. 7A−D, both wound-healing (Fig. 7A, B) and Transwell migration assays (Fig. 7C, D) revealed that the PAR1 antagonist SCH79797 but not the PAR2 antagonist FSLLRY-NH2 inhibited the KLK8-induced migration of RKO and SW480 cells. The invasiveness of CRC cells was also influenced by SCH79797. In RKO and SW480 cells, we observed that the number of invaded cells was decreased in SCH79797-treated cells (Fig. 7E, F).

These results demonstrated that the inhibition of PAR1 but not PAR2 could reverse the improved proliferative and metastatic potential, including migration and invasion, of CRC cells induced by KLK8 overexpression.

### PAR1 inhibition suppresses EMT in colorectal cancer cells induced by KLK8 in vitro

**Fig. 8:**
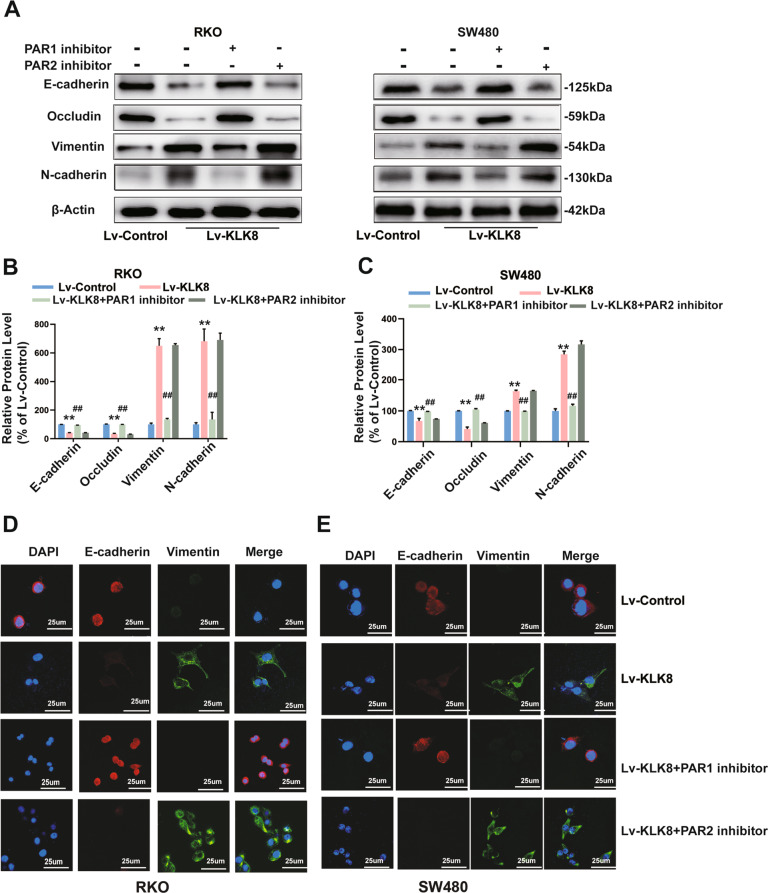
PAR1 inhibition suppresses EMT in CRC cells. **A** Western blotting analysis showed that PAR1 inhibition decreased the expression levels of the epithelial cell markers E-cadherin and occludin and increased the mesenchymal markers N-cadherin and vimentin in RKO and SW480 cells compared with the Lv-KLK8 group. **B**, **C** Quantification of the protein expression of E-cadherin, N-cadherin, vimentin and occludin represented in (**A**). **D**, **E** Immunofluorescence of E-cadherin and vimentin in RKO (**D**) and SW480 cells (**E**) treated with PAR1 antagonists SCH79797 and PAR2 antagonist FSLLRY-NH2. All of the data are presented as the mean ± SEM from three independent experiments. **P < 0.01 vs. Lv-control; ^##^*P* < 0.01 vs. Lv-KLK8.

To explore whether PAR inhibition suppresses CRC metastasis via EMT, we detected the EMT index in RKO and SW480 cells exposed to the PAR1 and PAR2 antagonists or controls. As shown in Fig. 8A−C, E-cadherin and occludin were significantly decreased in KLK8-overexpressing RKO and SW480 cells. N-cadherin and vimentin were markedly increased in KLK8-overexpressing RKO and SW480 cells. Conversely, pretreatment with the PAR1 antagonist SCH79797 reversed this effect (Fig. 8A−C). Immunofluorescence of E-cadherin and vimentin in RKO and SW480 cells treated with the PAR1 antagonist SCH79797 showed the same result (Fig. 8D, E). SCH79797 reversed the decreased expression of E-cadherin and increased expression of vimentin caused by KLK8 overexpression and inhibited EMT, while FSLLRY-NH2 had no effect.

### PAR1 inhibition suppresses proliferation and metastasis in colorectal cancer cells induced by KLK8 in vivo

Based on the results described above, it is clear that KLK8 promoted CRC metastasis in vitro, which could be abolished by the PAR1 antagonist SCH79797. Additionally, to establish whether KLK8 and PAR activity was related to proliferation and metastasis in vivo, we developed BALB/c nude mouse subcutaneous xenograft and metastatic models by injecting KLK8-overexpressing RKO cells and treating them with a PAR1 inhibitor.

**Fig. 9:**
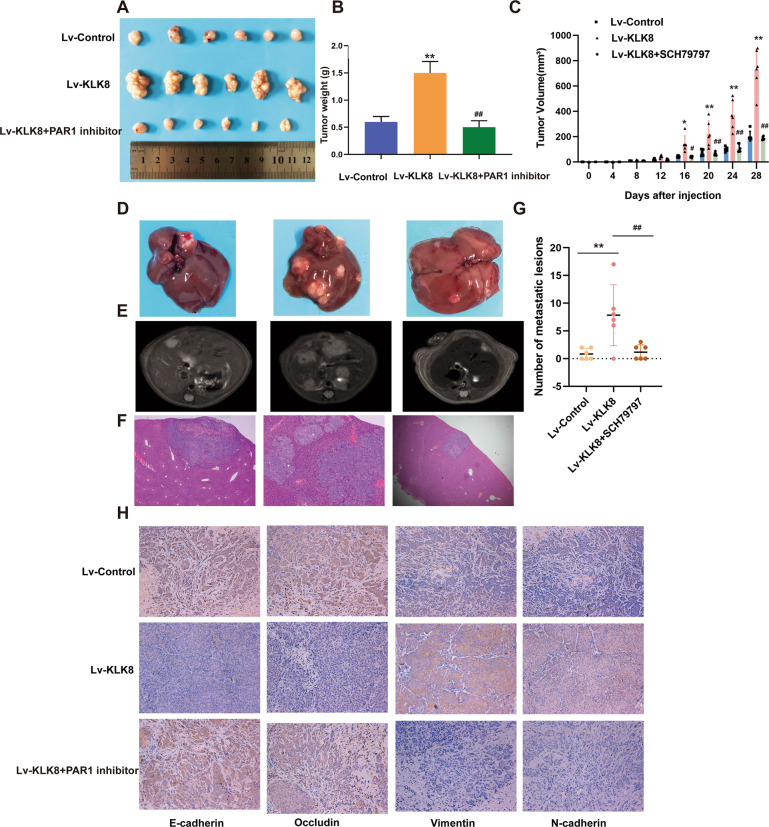
PAR1 inhibition suppresses proliferation and metastasis in colorectal cancer cells induced by KLK8 in vivo. **A** Photographs of tumours excised from mice injected with normal cells or cells with modified KLK8 expression (*n* = 6 per group). After 28 days, the mice were sacrificed, and the tumours were weighed (**B**). **C** KLK8-overexpressing cells produced larger tumour masses than control cells. **D** Representative images of livers after injection of modified RKO cells into the spleen. The metastatic nodules in the Lv-KLK8 group is clearly shown. **E** Representative images of PET CT and H&E staining (**F**) of metastatic nodules in the livers, ×100. **G** The numbers of metastatic nodules in the livers. **H** Immunohistochemical staining for E-cadherin, N-cadherin, vimentin and occludin expression in harvested mouse tumour samples, ×200. The statistical analyses are shown (*n* = 6). *P < 0.05, **P < 0.01 vs. Lv-control; ^#^*P* < 0.05, ^##^*P* < 0.01 vs. Lv-KLK8.

As shown in Fig. 9A−C, we observed that the tumour weights and volumes obtained with KLK8-overexpressing cells were larger and the weights of the mice were lower than those obtained with the control cells, while the tumour volume in the PAR1 inhibition group was smaller than that in the KLK8 overexpression group (Fig. 9A−C). These results indicated that the overexpression of KLK8 led to the promotion of tumour growth, while treatment with the PAR1 inhibitor SCH79797 reversed the accelerated tumour growth in vivo.

Furthermore, we also achieved consistent results in in vivo metastasis assays. After the mice were euthanized at 4 weeks, livers were obtained as shown in Fig. 9D. The livers of the metastatic mice in the KLK8 overexpression group were filled with more metastatic nodules than the control mice, whereas the group that received the PAR1 inhibitor SCH79797 treatment exhibited fewer metastatic lesions. Imaging analysis (Fig. 9E) and H&E staining (Fig. 9F) of the livers from the group that received KLK8-overexpressing RKO cells also showed greater tumour metastasis than those from the group that received SCH79797 treatment. Liver metastasis was found in 83.3% (5/6) of mice in the Lv-KLK8 group compared with 50% (3/6) in the Lv-Control group and 50% (3/6) in the SCH79797-treated group (Fig. 9G).

To assess EMT activity in metastatic models, we performed E-cadherin, N-cadherin, vimentin and occludin immunohistochemical staining on sections of metastatic tissues. Decreased expression of E-cadherin and occludin and increased expression of vimentin and N-cadherin were observed in the KLK8-overexpression group compared to the control group, while treatment with the PAR1 antagonists reversed this effect (Fig. 9H).

In conclusion, these data are consistent with the results of the in vitro cell proliferation, migration and invasion assays and indicate that KLK8 is associated with CRC progression and tumorigenesis in vivo, and these phenomena could be reversed by PAR1 inhibition.

## Discussion

Human kallikrein-related peptidases (KLKs), present in various human tissues, are a group of serine proteases encoded by a multigene family tightly clustered on chromosome *19q13.4* [6–8, 35]. Recently, KLKs have emerged as regulators of cancer growth, invasion and metastasis [36, 37]. For example, overexpression of KLK6 was found to promote gastric cancer cell growth, migration and invasion in vitro [38]. Higher expression of KLK family members 5–8 (KLK5–8) was associated with a more aggressive clinicopathologic phenotype and worse prognosis in endometrial cancer (EC) patients [39]. In contrast, low expression of KLK2 was associated with advanced tumour stage, more lymph node metastasis, increased cell proliferation, positive resection margin, and early PSA recurrence in prostate cancer [40]. However, the role of KLKs in CRC remains largely unknown. In this study, we investigated the expression profile of KLKs in CRC via TCGA and GTEx databases and found that KLK1, KLK6, KLK8, KLK10, KLK11 and KLK12 were highly expressed in CRC tissues compared to normal tissues. Furthermore, it was found that only KLK6, KLK8 and KLK10 predicted poor prognosis in CRC. Since previous studies have reported the clinical significance and underlying mechanisms of KLK6 [10–12] and KLK10 [13–15] in CRC [16–18], we focused on the significant role of KLK8 in CRC.

KLK8 has been considered a biological marker in several malignant tumours. Abnormal expression of KLK8 is associated with both favourable and unfavourable prognosis [41]. For example, elevated expression of KLK8 is observed in ovarian cancer [42], oral squamous cell carcinoma [43], salivary gland tumours [44] and cervical cancer [45] and indicates unfavourable prognosis in lung cancer [46] and breast cancer [47]. However, KLK8 upregulation predicts favourable prognosis in non-small cell lung cancer [48] and ovarian cancer [49]. Using two CRC cell lines, RKO and SW480 cells, a previous study [50] and the present study indicated that KLK8 could facilitate the proliferation, migration and invasion of CRC cells in vitro. In addition, using the xenograft model, the present study found that the tumour volume of the mice in the Lv-KLK8 group was much higher than that of the mice in the Lv-Control group. In the metastatic mouse model, mice in the Lv-KLK8 group exhibited more metastatic lesions than those in the Lv-Control group. These results demonstrated that KLK8 promoted the growth and metastasis of CRC in vivo.

CRC ranks third in terms of morbidity but second in terms of mortality, largely because of the extensive invasion and metastasis of the tumour [1, 2, 51]. EMT refers to the transition of epithelial cells into mesenchymal phenotype cells, which causes a high capacity for tumour migration and invasion [23, 52, 53]. A previous study reported that decreased expression of KLK6 in oral squamous cell carcinoma (OSCC) stimulated EMT, leading to enhanced migration and invasion in vitro [54]. However, whether other KLKs are involved in EMT remains largely unknown. Using GSEA, we found that high KLK8 expression was positively related to EMT. Western blotting and immunofluorescence assays showed that KLK8 overexpression led to decreases in the epithelial markers E-cadherin and occludin and increases in the mesenchymal markers vimentin and N-cadherin in RKO and SW480 cells. Moreover, in metastatic mouse models, the livers of mice in the Lv-KLK8 group exhibited decreased expression of E-cadherin and occludin and increased expression of vimentin and N-cadherin. Collectively, these results provide the first in vitro and in vivo evidence that KLK8 contributes to the development of EMT in CRC.

Effects of KLK8 on proliferation and invasion of CRC cells are somewhat similar to those of KLK6 and KLK10. Mechanistically, KLK10 silencing is found to attenuate the progression of CRC by inhibiting cell growth and glycolysis via the PI3K/AKT/mTOR signalling [16]. As for KLK6, Chen et al. [55] reported that the active KLK6 induces phosphorylation of SMAD 2/3 proteins and promotes EMT. To identify functional miRNA−mRNA interactions associated with KLK6-mediated invasiveness of colon cancer, Sells et al. [56] performed the integrated miRNA and mRNA expression profiling, and indicated that the established miRNA−mRNA interactions modulate cellular proliferation, differentiation and EMT in KLK6-expressing colon cancer cells via the TGF-β signalling pathway and RAS-related GTP-binding proteins. KLK8 is known to cleave the extracellular portion of several membrane proteins, such as PARs, neuregulin-1, synaptic adhesion molecule L1, Ephrin type-B receptor 2, pro-epidermal growth factor (pro-EGF), and kininogens. Of all the substrate proteins that could be cleaved by KLK8, PAR1 and PAR2 have been implicated in the development of CRC [24, 57–59]. The present study provided evidence that PAR1 but not PAR2 contributed to KLK8-induced CRC cell proliferation, migration and EMT in vitro. Pharmacological inhibition of PAR1 also suppressed KLK8-induced CRC progression and tumorigenesis in vivo. These novel findings suggest a potential PAR1 targeted therapeutic strategy in CRC patients with high KLK8 expression.

EMT takes centre stage during the progression of degenerative fibrotic diseases and cancer [19, 34, 60–63]. The EMT process is controlled by a network of signalling pathways such as transforming growth factor (TGF)-β/Smad and Hippo pathways, and is executed by a subset of transcription factors including ZEB1/2, Snail/Slug, Twist, and YAP/TAZ [28, 61, 64–69]. Previous studies have reported that PAR-1 activation contributes to renal EMT and interstitial fibrosis during chronic obstructive nephropathy [70, 71]. Pharmacological inhibition of PAR-1 provides a renoprotective strategy by virtue of its anti-fibrotic effects, mediated partly via inhibition of Smad-dependent TGF-β signalling [71]. In gastric and CRC cells, PAR-1 activation leads to upregulation of pro-EMT transcriptional factor Snail and Twist, respectively [28, 67]. PAR-1 is also involved in the activation of YAP/TAZ [64, 65]. Inhibition of PAR-1 suppresses YAP/TAZ-induced EMT, invasion, migration, cancer stem cell-like properties, tumour growth and metastasis of breast cancer cells [65]. Notably, a recent study demonstrates that upregulation of KLK8 may account for the elevated TGF-β/Smad signalling and pro-fibrotic transcription factors including ZEB1/2, Snail/Slug, and Twist in the context of diabetic cardiac fibrosis [66]. Whether these signalling pathways and transcription factors are involved in KLK8/PAR1-driven EMT in CRC cells merits future investigation.

As the earliest and most in-depth molecule of the PAR family member, PAR-1 has been shown to have protumorigenic effects and is an emerging anticancer drug target [57]. The application of the PAR1 inhibitor PZ-128 as an anti-metastatic and anti-angiogenic therapeutic agent in melanoma and breast, ovarian, and lung cancer is being reviewed [57, 72–74]. In our study, inhibition of PAR1 reversed the protumorigenic effect of KLK8 both in vivo and in vitro in CRC. Whether the use of PAR-1 inhibitors may have the effects of inhibiting colorectal tumour growth and reducing metastasis in the clinic is still of interest. Thus, PAR-1-centred studies need to be further enriched and assessed, not only to elucidate its protumorigenic functions but also to study its use as a promising target for clinical therapy.

In conclusion, KLK8 was upregulated in CRC and predicted poor prognosis in CRC patients. Furthermore, KLK8 facilitated the proliferation, migration, invasion and EMT of CRC cells both in vitro and in vivo. Moreover, the present study showed for the first time that KLK8 exerted its protumorigenic effects via a PAR1-dependent mechanism. These findings provide a helpful theoretical basis for KLK8/PAR1-based therapeutic strategies for the treatment of CRC.

## Supplementary information

supplementary materials

## Data Availability

The data that support the findings of this study are available from the corresponding authors upon reasonable request.
