# Supplementary material for: KLK8 promotes the proliferation and metastasis of colorectal cancer via the activation of EMT associated with PAR1
Source: Cell Death Dis. 2021 Sep 22;12(10):860. doi: 10.1038/s41419-021-04149-x (PMC8458432; doi:10.1038/s41419-021-04149-x)
Supplement: Supplementary file 1 — supplementary materials [file 41419_2021_4149_MOESM1_ESM.pdf]

Supplemental Digital Content

Supplement figures

Fig. 1 The role of KLKs in the CRC. **A** The expression of KLKs in CRC tissues (n=275) and normal tissues (n=349) analyzed in TCGA and GTEx databases. **B, C** Disease-free survival (DFS) (B)and Overall survival (OS) (C) were compared between patients with low and high KLKs expression in the GSE39582 database. *, P < 0.05; **, P < 0.01; *** P < 0.001.

Fig. 2 **A-D** Relative expression level of KLK8 in normal and tumor tissues (both colon and rectal cancer compared with normal tissue) from 4 independent colorectal datasets in the Oncomine database.

Fig. 3 **A-B** Western blot analysis of the expression of KLK8 after treatment with Lv-KLK8(**A**) and KLK8 siRNA (**B**) in RKO and SW480 cells. β-actin was used as an internal control.

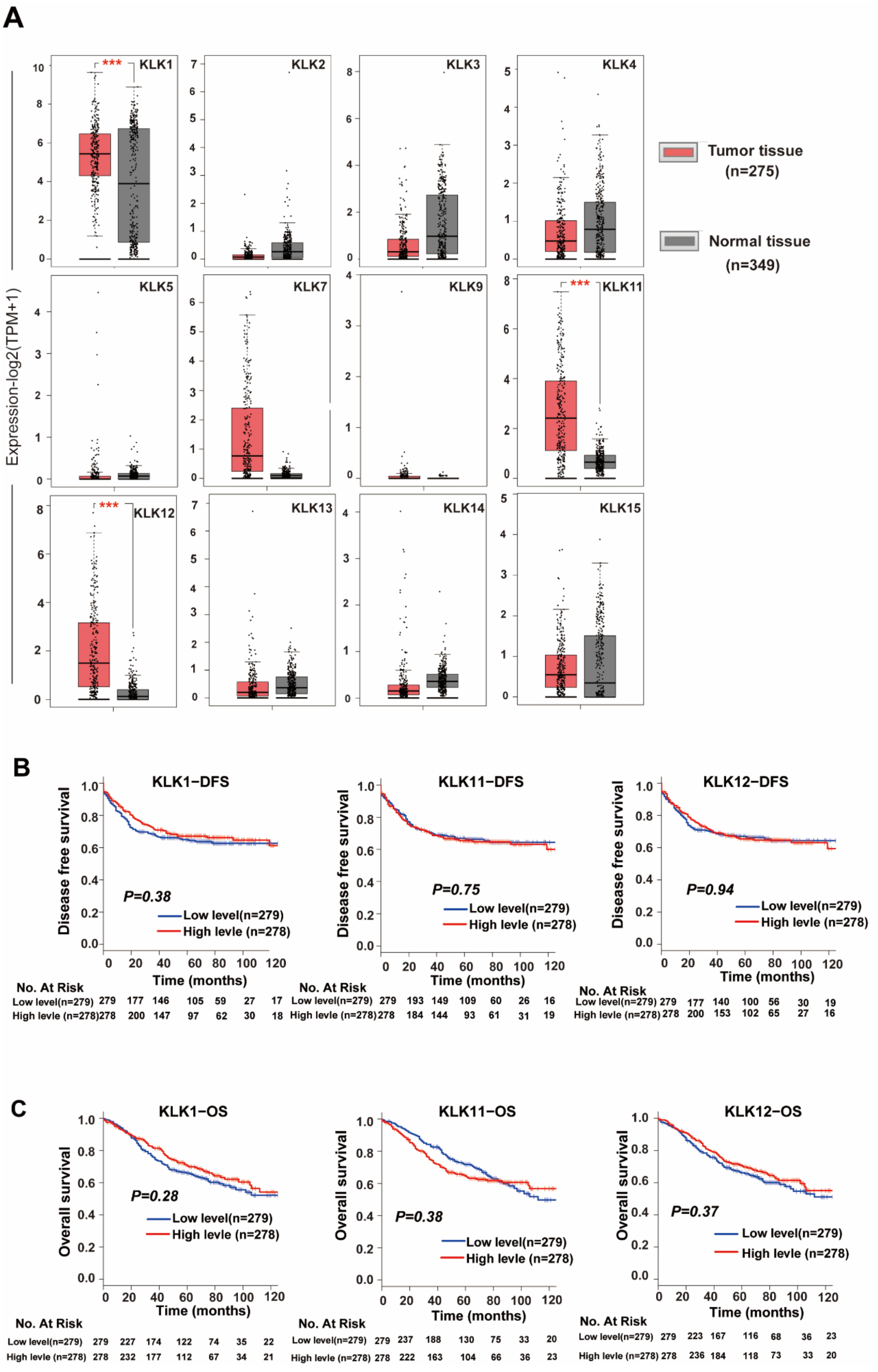

Supplement fig. 1 The role of KLKs in the CRC.

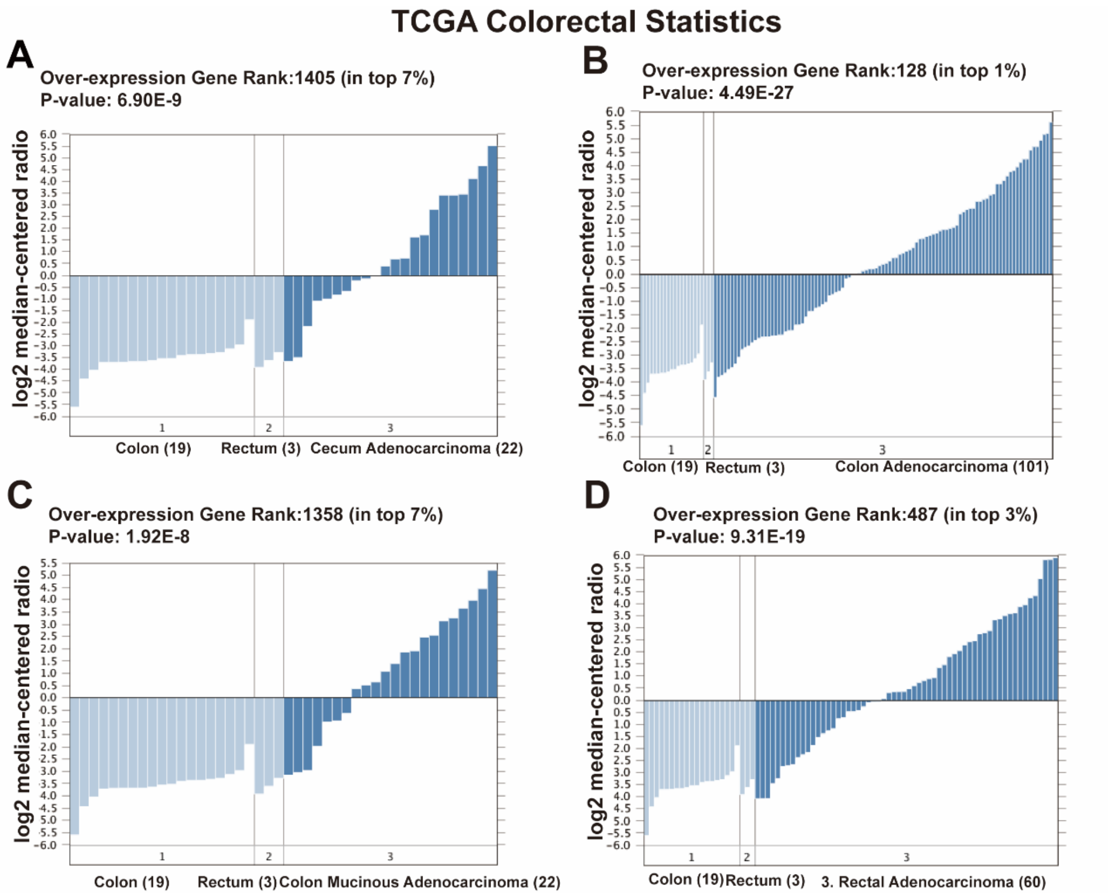

Supplement fig. 2 **A-D** Relative expression level of KLK8 in normal and tumor tissues (both colon and rectal cancer compared with normal tissue) from 4 independent colorectal datasets in the Oncomine database.

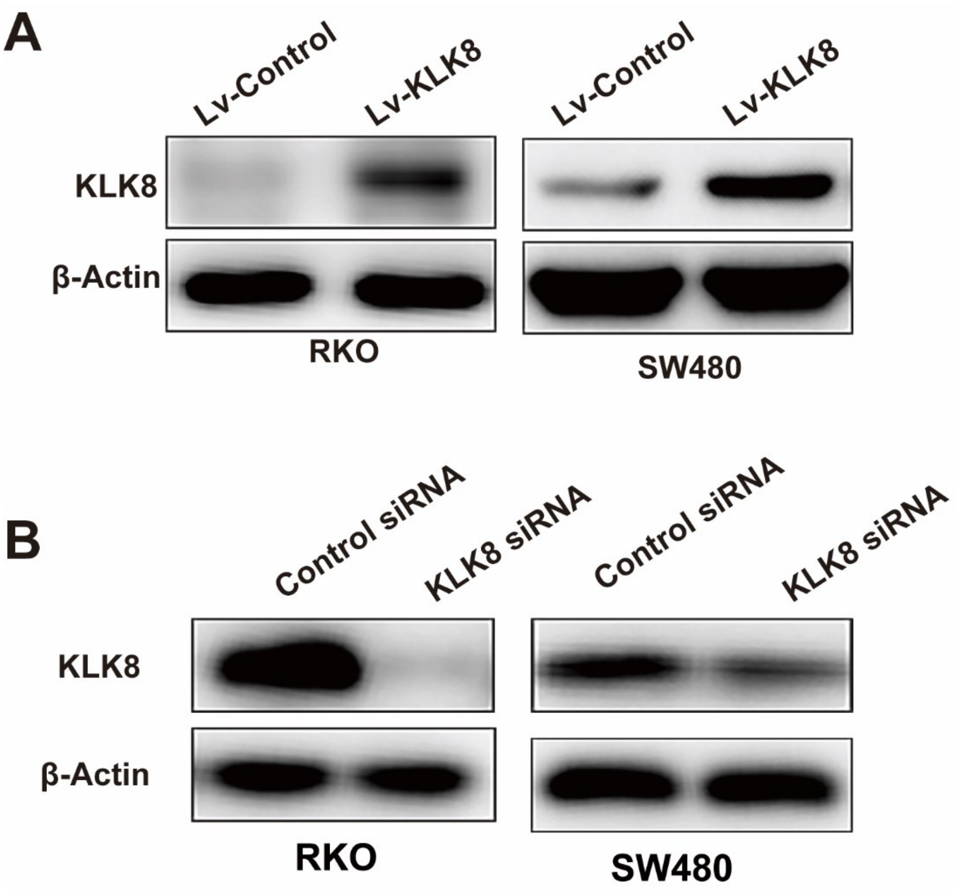

Supplement fig. 3 Western blot analysis of the expression of KLK8 after treatment with Lv-KLK8 or KLK8 siRNA in RKO and SW480 cells. β-actin was used as an internal control.

Ethics approval and consent to participate

All research and procedures involving human subjects were approved by the ethics committee of Fudan University Shanghai Cancer Center. The methods in this study were carried out in accordance with the approved guidelines by Fudan University Shanghai Cancer Center.

Ethics Statement

This study was approved by the Ethics Committee of Fudan University Shanghai Cancer Center. (Shanghai, China).

Consent for publication

We would like to submit the enclosed manuscript entitled 'KLK8 promotes the proliferation and metastasis of colorectal cancer via the activation of EMT associated with PAR1', which we wish to be considered for publication in 'Cell death & Disease'. Manuscript is approved by all authors for publication. All the authors listed have approved the manuscript that is enclosed.

Availability of data and materials

None

Competing interests

The authors declare that they have no conflict of interest

Funding statement

This work was supported by grants from National Natural Science Foundation of China (No. 81471852, No. 31671213, No. 31871156) and Shanghai Natural Science Foundation Program (20ZR1412900).

Authors' Contributions Statement

Qing Hua was responsible for conducting the study, under the supervision of Zhirong Sun, Xiaoyan Zhu and Pingbo Xu and contributed to the experimental design; Qing Hua, Zhirong Sun and Yi Liu did the experiments and analyzed the data. Xuefang Shen and Weiwei Zhao provided clinical samples and analysed the data. Qing Hua drafted the paper, which was revised by Xiaoyan Zhu and Pingbo Xu. All authors read and approved the final manuscript.

Acknowledgments

We appreciated two expert pathologists, Dr Yue Zhang and Min Ye for data interpretation of the H&E and IHC staining images of clinical samples.
